# Directions to Army Surgeons on the Field of Battle

**Published:** 1861-08

**Authors:** G. J. Guthrie

**Affiliations:** Surgeon-General to the British Forces during the Crimean War


### SELECTED.

### DIRECTIONS TO ARMY SURGEONS ON THE FIELD OF BATTLE.

BY G. J. GUTHRIE,

*Surgeon-General to the British Forces during the Crimean War.*

[From his Pamphlet on the Hospital Brigade.]

1. Water being of the utmost importance to wounded men, care should be taken when before the enemy, not only that the barrels attached to the conveyance-carts are properly filled with good water, but that skins for holding water, or such other means as are commonly used in the country for carrying it, should be procured and duly filled.

2. Bandages or rollers, applied on the field of battle are, in general, so many things wasted, as they become dirty and stiff, and are usually cut away and destroyed, without having been really useful; they are therefore not forthcoming when required, and would be of no use.

3. Simple gun-shot wounds require nothing more, for the first two or three days, than the application of a piece of wet or oiled linen, fastened on with a strip of sticking-plaster, or, if possible, kept constantly wet and cold with water. When cold disagrees, warm water should be substituted.

4. Wounds made by swords, sabres, or other sharp-cutting instruments, are to be treated principally by position. Thus, a cut down to the bone, across the thick part of the arm, immediately below the shoulder is to be treated by raising the arm to or above right angle with the body, in which position it is to be retained, however inconvenient it may be. Ligatures may be inserted, but through the skin only. If the throat be cut across in front, any great vessels should be tied, and the oozing stopped by a sponge. After a few hours, when the oozing is arrested, the sponge should be removed, and the head brought down towards the chest, and retained in that position without ligatures; if this is done too soon, the sufferer may possibly be suffocated by the infiltration of blood into the areolar tissue of the parts adjacent.

5. If the cavity of the chest is opened into by a sword or lance, it is of the utmost importance that the wound in the skin should be effectively closed, and this can only be done by sewing it up as a tailor or a lady would sew up a seam, skin only being included; a compress of list should be applied over the stitches, fastened on by sticking plaster. The patient is then to be placed on the wounded side, that the lung may fall down, if it can, upon, or apply itself to the wounded part, and adhere to it, by which happy and hoped-for accident life will in all probability be preserved. If the lung should be seen protruding in the wound, it should not be returned beyond the level of the ribs, but be covered over by the external parts.

6. It is advisable to encourage previously the discharge of blood from the cavity of the chest, if any have fallen into it; but if the bleeding from within should continue, so as to place the life of the sufferer in danger, the external wound should be closed, and events awaited.

7. When it is doubtful whether the bleeding proceeds from the cavity of the chest, or from the intercostal artery (a surgical bugbear), an incision through the skin and the external intercostal muscle will expose the artery close to the edge of the rib having the internal intercostal muscle behind it. The vessel thus exposed may be tied, or the end pinched by the forceps, until it ceases to bleed. Tying a string round the ribs is a destructive piece of cruelty, and the plugs, &c., formerly recommended, may be considered as surgical incongruities.

8. A gun-shot wound in the chest cannot close by adhesion, and must remain open. The position of the sufferer should therefore be that which is most comfortable to him. A small hole penetrating the cavity is more dangerous than a large one, and the wound is less dangerous if the ball goes through the body. The wounds should be examined and enlarged, if necessary, in order to remove all extraneous substances, even if they should be seen to stick on the surface of the lung if the opening should be covered with soft oiled or wet lint—a bandage when agreeable. The ear of the surgeon and the stethoscope are invaluable aids, and ought always to be in use; indeed, no injury of the chest can be scientifically treated without them.

9. Incised and gun-shot wounds of the abdomen are to be treated in *nearly* a similar manner; the position in both being that which is most agreeable to the patient, the parts being relaxed.

11. In wounds of the bladder, an elastic catheter is generally necessary. If it cannot be passed an opening should be made in the perinæum for the evacuation of the urine, with as little delay as possible.

12. In gun-shot fractures of the skull, the loose broken pieces of bone, and all extraneous substances, are to be removed as soon as possible, and depressed fractures of bone are to be raised. A deep cut made by a heavy sword through the bone into the brain, generally causes a considerable depression of the inner table of the bone, whilst the outer may appear to be merely divided.

13. An arm is rarely to be amputated, except from the effects of a cannon-shot. The head of the bone is to be sawn off, if necessary. The elbow-joint is to be cut out, if destroyed, and the sufferer, in either case, may have a very useful arm.

14. In a case of gun-shot fracture of the upper arm, in which the bone is much splintered, incisions are to be made, for the removal of all the broken pieces which it is feasible to take away. The elbow is to be supported. The forearm is to be treated in a similar manner; the splints used should be solid.

15. The hand is never to be amputated, unless all or nearly all its parts are destroyed. Different bones of it and of the wrist are to be removed when irrecoverably injured, with or without the metacarpal bones and fingers or the thumb; but a thumb and one finger should always be preserved when possible.

16. The head of the thigh bone should be sawn off when broken by a musket-ball. Amputation at the hip-joint should only be done when the fracture extends some distance into the shaft, or the limb is destroyed by cannon-shot.

17. The knee-joint should be cut out when irrecoverably injured; but the limb is not to be amputated until it cannot be avoided.

18. A gun-shot fracture of the middle of the thigh, attended by great splintering, is a case for amputation. In less difficult cases, the splinters should be removed by incisions, particularly when they can be made on the upper and outer side of the thigh. The line should be placed on a straight, firm splint. A broken thigh does not admit of much, and sometimes of no extension, without an unadvisable increase of suffering. An inch or two of shortening in the thigh does not so materially interfere with progression as to make the sufferer regret having escaped amputation.

19. A leg injured below the knee should rarely be amputated in the first instance, unless from the effects of a cannon-shot. The splinters of bone are all to be immediately removed, by saw or forceps, after due incisions. The limb should be placed in iron splints, and hung on a permanent frame, as affording the greatest comfort, and probable chance of ultimate success.

20. An ankle-joint is to be cut out, unless the tendons around are too much injured, and so are the tarsal and metatarsal bones and toes. Incisions have hitherto been too little employed in the early treatment of these injuries of the foot for the removal of extraneous substances.

21. A wound of the principal artery of the thigh, in addition to a gun-shot fracture, renders immediate amputation necessary. In *no other part* of the body is amputation to be done in the first instance for such injury. Ligatures are to be placed on the wounded artery, one above, the other below the wound, and events awaited.

22. The occurrence of mortification in any of these cases will be known by the change of color in the skin. It will rarely occur in the upper extremity, but will frequently do so in the lower. When about to take place, the color of the skin of the foot changes, from the natural flesh color to a tallowy or mottled white. Amputation should be performed immediately above the fractured part. The mortification is yet local.

23. When this discoloration has not been observed, and the part shrinks, or gangrene has set in with more marked appearances, but yet seems to have *stopped* at the ankle, delay is, perhaps, admissible, but if it should again spread, or its cessation be doubtful, amputation should take place forthwith, although under less favorable circumstances. The mortification is becoming, or has become constitutional.

24. Bleeding, to the loss of life, is not a common occurrence in gun-shot wounds, although many do bleed considerably, seldom, however, requiring the application of a tourniquet as a matter of necessity although frequently as one of precaution.

25. When the great artery of the thigh is wounded (not torn across), the bone being *uninjured*, the sufferer will probably bleed to death, unless aid be afforded, by making compression above, and on the bleeding part. A long, but not broad stone, tied sharply on with a handkerchief, will often suffice until assistance can be obtained, when both ends of the divided or wounded artery are to be secured by ligatures.

26. The upper end of the great artery of the thigh bleeds scarlet blood, the lower and dark venouscolored blood; and this is not departed from in a case of accidental injury, unless there have been previous disease in the limb. A knowledge of this fact or circumstance, which continues for several days, will prevent a mistake at the moment of injury, and at a subsequent period, if secondary hæmorrhage should occur. In the *upper* extremity both ends of the principal artery bleed scarlet blood, from the free collateral circulation, and from the anastomoses in the hand.

27. From this cause, mortification rarely takes place after a wound of the principal artery of the arm, or even of the arm-pit. It *frequently* follows a wound of the principal artery in the upper, middle, or even lower parts of the thigh, rendering amputation neccessary.

28. It is a great question, when the bone is *uninjured*, where, and at what part, the amputation should be performed. Mortification of the foot and leg, from such a wound, is disposed to stop a little below the knee, if it should not destroy the sufferer; and the operation, if done in the first instance, as soon as the tallowy or mottled appearance of the foot is observed, should be done at that part; the wound of the artery, and the operation for securing the vessel above and below the wound, being left unheeded. By this proceeding when successful the knee-joint is saved, whilst an amputation above the middle of the thigh is always very doubtful in its result.

29. When mortification has taken place from any cause, and has been arrested below the knee, and the dead parts show some sign of separation, it is usual to amputate above the knee. By not doing it, but by gradually separating and removing the dead parts, under the use of disinfecting medicaments and fresh air, a good stump may be ultimately made, the knee-joint and life being preserved, which latter is frequently lost after amputation under such circumstances.

30. Hospital gangrene, when it unfortunately occurs, should be considered to be contagious and infectious, and is to be treated locally by destructive remedies, such as nitrid acid, and the bivouacking or encamping of the remainder of the wounded, if it can be effected, or their removal to the open air.

31. Poultices have been very often applied in gun-shot wounds, from laziness, or to cover neglect, and should be used as seldom as possible.

32. Chloroform may be administered in all cases of amputation of the upper extremity and below the knee, and in all minor operations; which cases may also be deferred, without disadvantage, until the more serious operations are performed.

33. Amputation of the upper and middle parts of the thigh are to be done as soon as possible after the receipt of the injury. The administration of chloroform in them, when there is much prostration, is doubtful, and must be attended to, and observed with great care. The question whether it should or should not be administered in such cases being undecided.

34. If the young surgeon should not feel quite equal to the ready performance of the various operations recommended, many of them requiring great anatomical knowledge and manual dexterity (and it is not to be expected that he should), he should avail himself of every opportunity which may offer of perfecting his knowledge.—*Lancet.*

